# Tubercular Kidneys

**Published:** 1869-04-01

**Authors:** 


### ORGIINAL COMMUNICATIONS.

### *Tubercular Kidneys.*

Dec. 5, 1864.—E. K———, female, aged about thirty-five years. Much emaciated and prostrated; vomiting ingesta persistently. Complains of severe pain over the left kidney; tongue slightly reddened, pulse and skin nearly normal. *No cough*; bowels obstinately constipated. No satisfactory history of the case can be acquired. Directed a blister to be applied over the left lumbar region, and the urine to be reserved for examination.

Dec. 6th.—Renal pain greatly relieved. Urine nearly neutral (very faintly acid). Specific gravity 1025 (at 65° Fr.). No perceptible change was produced by any chemical reagent, except the deposit of a brown, flocculent precipitate, upon boiling it after the addition of nearly an equal bulk of liquor potassæ. Under the microscope a small quantity of finely granular matter was visible, intermingled with a very few cells obscurely resembling pus cells.

The renal pain, as stated, was diminished, but the gastric uneasiness and tenderness appeared to have increased.

Ordered one-fourth of a grain of calomel every three hours! which, producing no amelioration of her condition, after the lapse of forty-eight hours, was discontinued. She was then confined exclusively to milk, of which about two ounces were administered hourly. This was, like every thing else, rejected by the stomach, and the patient sank, apparently from inanition, on the 12th (seven days). *The secretion of urine amounted to about one quart daily*, maintaining its characteristics as already described. The bowels were obstinately constipated.

*Sectio cadaveris* eight hours after death. *Head*, not examined. *Body*, emaciated to an extreme degree; subcutaneous layer of fat of a deep orange color. *Chest*, pleural surfaces adherent throughout their whole extent; right lung emphysematous, left lung collapsed. *Structure, normal.* Heart, pericardium containing about one ounce of serum; right ventricular and auricular walls fatty throughout about one-third of their thickness.

A firm, white, old fibrinous clot, one and one-half inches in length by half an inch in thickness, in right ventricle. Left ventricle concentrically hypertrophied; walls about one and a quarter inches in thickness; valves normal.

Liver much engorged with venous blood, but otherwise apparently healthy. Gall-bladder distended with bile. Spleen enlarged, engorged with blood, but otherwise apparently healthy, except upon its anterior face, which had undergone complete cartilaginous degeneration for the space of four inches in length, two in breadth, and one-sixth of an inch in thickness. Right kidney reduced to little more than half its normal size, lobulated externally, and of a deep purple color, its interior converted entirely into tubercular matter, except the pelvis, which was occupied by a mass of fat. Left kidney enlarged to twice its normal size, of a deep purple color externally, occupied internally, wholly by vomicæ, enclosed by dense, tough tissue, varying in size from that of a pin's head to that of a hen's egg, stuffed with tubercular matter. The lower extremity, for the space of two inches in breadth by half an inch in depth, was healthy, constituting the whole of the secreting structure remaining in either organ.

The mucous membrane of the stomach and intestinal canal throughout was congested, but otherwise healthy, the intestines being in many places adherent from old circumscribed peritonitis.

The above reported case may possibly interest the readers of The Journal, from its rarity, and from the absence of objective symptoms of sufficient pathognomic value to serve as a basis for a diagnosis.

The striking peculiarities are:

1st.—The existence of large masses of tubercle entirely isolated, limited exclusively to one organ, or system.

2d.—The maintenance of the functional energy of the kidney to a degree approximating so nearly to the normal standard; so small a portion of its structure remaining intact, involving,

3d.—The absence of constitutional indications (aside from the gastric irritation, if that could be so considered) of retention of the elements of the urinary secretion in the blood.

4th.—The absence, from the urinary secretion itself, of any sufficient indications of the true condition of the secreting organs. Having had many opportunities of examining tubercular sputa, and also urinary deposits, with the microscope, the existence, in this case, of tubercular matter in the urine in appreciable quantity would surely have been recognized.

An accurate diagnosis, in this instance, could possess only a scientific value, having no practical significance whatever, and yet the physician can be placed in few more embarrassing positions than to find himself utterly unable to determine the true pathological condition of his patient.

